# Crisis on the game board – a novel approach to teach medical students about disaster medicine

**DOI:** 10.3205/zma001192

**Published:** 2018-11-15

**Authors:** Simon Drees, Karin Geffert, Rex Brynen

**Affiliations:** 1 German Medical Students' Association, Berlin, Germany; 2 Charité – Universitätsmedizin Berlin, Berlin, Germany; 3 Julius-Maximilians Universität Würzburg, Medical Faculty, Würzburg, Germany; 4 McGill University, Department of Political Science, Montreal, Canada

**Keywords:** Disasters, Disaster Medicine, Relief Work, Simulation Training, Medical Education

## Abstract

**Introduction: **Every year, natural and other disasters cause significant loss of life around the world. This calls for an improved response from, among others, the health professions to strengthen disaster medicine, whether relating to prevention, emergency response or recovery. It includes both knowledge and competencies, such as planning, coordination, and communication. Simulations can be used to acquire these competencies.

**Project description:** In 2016, the German Medical Students’ Association founded the project “Disaster Medicine” with the goal of educating and connecting medical students interested in the topic. AFTERSHOCK, a board game simulating early disaster response after an earthquake, was utilized for workshops. It highlights the need for interagency cooperation and the challenges of responding to disasters in dynamic and highly complex settings. Seven workshops were facilitated between October 2016 and December 2017. A survey was conducted to assess participant satisfaction and the design of the workshop.

**Results: **89 German medical students participated and 74 (83 %) responded with written evaluation. Students generally reported moderate to low levels of previous knowledge. The event produced very positive feedback, with participants overwhelmingly finding the simulation to be a useful or very useful way to learn about the challenges of humanitarian assistance and disaster relief. Qualitative feedback included requests for more theoretical background information and highlighted the need for small group sizes.

**Discussion and Conclusion: **Board games such as AFTERSHOCK are well-suited for medical education and enjoy high rates of acceptance among students. To ensure deeper and longer-term learning, they should be accompanied by theoretical coursework.

## Zusammenfassung

**Einleitung: **Jedes Jahr verursachen Katastrophen eine signifikante Anzahl von Todesfällen weltweit. Eine Stärkung der Katastrophenmedizin, unter anderem durch Gesundheitsfachkräfte, in den Bereichen Prävention, Notfalleinsätze als auch bei dem Wiederaufbau ist erforderlich. Hierzu zählen Kompetenzen und Wissen in den Feldern Planung, Koordination und Kommunikation. Simulationen können dazu genutzt werden, diese Kompetenzen zu erwerben.

**Projektbeschreibung: **Die Bundesvertretung der Medizinstudierenden in Deutschland e.V. hat 2016 das Projekt „Katastrophenmedizin“ gegründet um interessierte Studierende fortbilden und vernetzen zu können. Für die Workshops wurde das Brettspiel AFTERSHOCK genutzt, welches die Phase der unmittelbaren Katastrophenhilfe nach einem Erdbeben simuliert. Sieben Workshops wurden zwischen Oktober 2016 und Dezember 2017 durchgeführt. Eine Umfrage wurde durchgeführt, um die Zufriedenheit der Teilnehmenden und den Aufbau des Workshops zu evaluieren.

**Ergebnisse:** 89 Medizinstudierende nahmen an den Workshops teil und 73 (83 %) beteiligten sich an der Evaluation. Die Studierenden gaben allgemein ein mittleres bis niedriges Vorwissen an. Die Veranstaltung wurde positiv bewertet und ein Großteil der Studierenden empfand die Simulation als einen hilfreichen oder sehr hilfreichen Weg um die Herausforderungen von humanitärer Hilfe und Katastrophenhilfe kennenzulernen. Qualitatives Feedback umfasste unter anderem die Forderung nach mehr theoretischem Hintergrundwissen und einer kleineren Gruppengröße.

**Diskussion und Schlussfolgerung:** Brettspiele wie AFTERSHOCK sind für die medizinische Ausbildung gut geeignet und bei Studierenden sehr beliebt. Um einen umfassenderen und langanhaltenden Lerneffekt zu erreichen, sollten sie durch theoretische Kurse begleitet werden.

## Introduction

The number of disasters remains alarmingly high: Man-made and natural disasters are threatening the lives of individuals and whole populations all around the world [1]. Even in Germany, storms and terror attacks are a considerable threat and an increasingly globalized world poses ever greater challenges. These developments call for an improved response from all sectors, including the health professions, to further strengthen disaster medicine [2]. The German national competency-based catalogue of learning objectives for medicine (NKLM), which was adopted in 2015 by all German medical faculties, includes one sub-competency (17.1.5) and two associated learning objectives related to disaster medicine [http://www.nklm.de]. These cover both the theoretical basis of disaster medicine as well as the concept of triage, thus defining that every German medical student should acquire basic disaster medicine knowledge. Unfortunately, the topic remains underrepresented in medical education: A recent evaluation by Wunderlich et al. among German medical students found that the number of students trained in disaster medicine remains low despite their strong interest in this topic [3].

Disaster medicine can be defined as “the science that seeks to address the adverse health and medical effects associated with disasters (...), and includes prevention, emergency response, and the recovery needs of affected individuals and populations” [4]. It requires not only theoretical knowledge, but also process and procedure-based competencies. Acquiring these basic competencies in practice is for the most part neither feasible nor ethical. Therefore, simulations are an irreplaceable cornerstone in education on disaster medicine. Indicative of this, the Emergency Capacity Building Project – a collaborative initiative involving seven of the largest non-governmental organizations involved in global disaster response – undertook a major study of simulation-based training methods in this sector. This found that simulations are a powerful tool for both training and planning in a safe-to-fail learning environment, noting that “simulations permit humanitarian actors, organisations, and governments to make errors in a safe, controlled environment rather than when actual lives are at stake” [5].

One key characteristic of humanitarian assistance and disaster relief is the multiplicity of (often autonomous) actors, organizations and capabilities involved, and the challenge of coordinating these in an often chaotic and uncertain environment. This is especially true of major global or transnational humanitarian crises – for example, the 2004 Indian Ocean tsunami, the 2010 Haiti earthquake, or the ongoing Syrian or Yemeni civil wars – in which a multiplicity of international organizations, national governments, non-governmental organizations, local actors and others are involved, with no clear organizational hierarchy. Training personnel to operate in such contexts involves teaching coordination skills, such as an understanding of various stakeholder perspectives, flexibility, effective communication, prioritization, and identifying new approaches and synergies. These sorts of challenges can be built into a simulation, helping participants to both understand their importance and develop the personal and intellectual skills to respond effectively. Unrelated to the content of the simulation, practicing these soft skills might also contribute to general competencies specified in the NKLM’s chapters on physician’s roles as communicators, team members and managers (see chapters 7,8 and 10 respectively) [http://www.nklm.de].

As part of the project we report on, workshops on disaster medicine including a board game simulation were conducted with German medical students. The goal of these workshops was to provide an understanding of the afore-mentioned complexities as well as basic information on disaster medicine and humanitarian aid. We evaluated the satisfaction of participants and the design of the workshop.

## Project description

In 2016, the German medical students’ association founded the project “Disaster medicine” to explore the possibilities of simulation and to offer interested students a platform for networking and sharing knowledge as well as information on educational opportunities related to disaster medicine. The project was initiated and lead by Karin Geffert and Simon Drees. The first workshop that was developed consisted of an introduction on disaster medicine and a simulation. For the latter, a board game developed by Rex Brynen, called “AFTERSHOCK: A Humanitarian Crisis Game” was chosen [https://paxsims.wordpress.com/aftershock/ [cited 2017 Dec 8]].

AFTERSHOCK was designed from the outset as an educational resource to be used in teaching university students, humanitarian and military personnel, and government officials about the challenges of disaster response [6]. Set in the fictional country of Carana, the game is based on challenges encountered during the 2010 Haiti earthquake, with additional material drawn from other humanitarian emergencies. It was developed with input from subject matter experts, and play-tested extensively. AFTERSHOCK covers approximately three months of humanitarian operations, including both the initial emergency and a later period of early recovery. Because Carana is a fragile, conflict-affected country, relief and reconstruction efforts may also involve issues of social unrest and political instability, especially during the early recovery stage once the initial shock of the crisis has worn off. Key issues highlighted in the game include: needs assessment and aid prioritization; coordination across multiple actors (host country, foreign military, United Nations, NGOs), each with differing priorities; local self-help by disaster-affected populations; internal displacement; epidemics; secondary disasters (aftershocks, flooding); security and host country stability; logistics and supply routes; relief-to-recovery transitions; and media relations. With the support of National Defense University, a game expansion was later developed to add additional focus on gender issues in humanitarian aid. The game is usually played with a two-hour timer, both to fix its duration and create time pressures on participants analogous to a real crisis. 

Since it was first published in 2015, AFTERSHOCK has been used in a variety of university-level courses around the world; to train Canadian humanitarian personnel [7]; as part of a World Health Organization-supported course in communicable disease control in a humanitarian emergency [8]; for humanitarian assistance/disaster relief (HADR) training in the US military [9]; and for pre-deployment training of peacekeepers and CIVPOL (civilian police) personnel in Chile [10]. A game typically involves 4-12 players organized into four teams, although various techniques can be used to deliver the game to larger audiences. 

Feedback from the game in other settings has been very positive. Surveys of students at both McGill University and from the Canadian Disaster and Humanitarian Response Training Program have indicated that 85-90 % of participants found the game enjoyable or very enjoyable, over 95 % view the game as good or very good at illustrating issues related to humanitarian assistance and aid coordination, and well over 90 % recommend that AFTERSHOCK be used in future courses [for complete data, see https://paxsims.wordpress.com/aftershock/]. 

The game materials were acquired for the German medical students’ association’s disaster medicine project at a purchasing price of 99.99$ in 2017. Seven workshops were conducted at German medical schools (Charité – Universitätsmedizin Berlin, Goethe Universität Frankfurt, Universität des Saarlandes Homburg) and as part of events organized by the German Medical Students’ Association (National Conferences in Freiburg and Mainz) between October 2016 and December 2016. The Workshops were advertised via Facebook or the German Medical Students’ Association’s mailing lists. All workshops followed the same structure: 30 minutes of introduction on disaster medicine (terminology, relevant institutions, basics of disaster response) preceded the AFTERSHOCK simulation (120 to 150 minutes). Standardized slides were used for both the introduction and the explanation of core concepts of the simulation. Participants were randomly assigned to the four teams at the start of the simulation. All workshops were concluded with a moderated debriefing that highlighted group dynamics as well as lessons learned and limitations of the AFTERSHOCK game (15 to 30 minutes). Only one facilitator was needed to conduct the workshops as described, but having a second facilitator to assist with game setup, answer questions during the simulation and moderate the debriefing proved to be helpful.

A paper-based evaluation form consisting of Likert scale-like questions on previous experience and satisfaction with the workshop’s structure, content and complexity as well as a free-text question regarding suggestions for improvement was distributed to participants at the end of the event. The items were based on previous AFTERSHOCK-evaluations mentioned above. A five-level scale was chosen in order to allow participants to express a neutral/undecided position (depending on the item). Evaluation data was analyzed using IBM SPSS Statistics 24.

## Results

A total of 89 participants attended the workshops (9-16 per workshop), 74 (83%) filled out the evaluation form (see attachment 1 ). All participants were medical students except for one nurse. Medical students had been studying for a mean of 6 semesters (range 1-13). 56.8% had previously attended an event organized by the German Medical Students’ Association. Among the participants, 17.6% rated their prior knowledge of humanitarian assistance as very low, 33.8% as low, 40.5% and neither high nor low, and 8.1% as high. None assessed a very high level of prior knowledge. 

**Figure 1:**
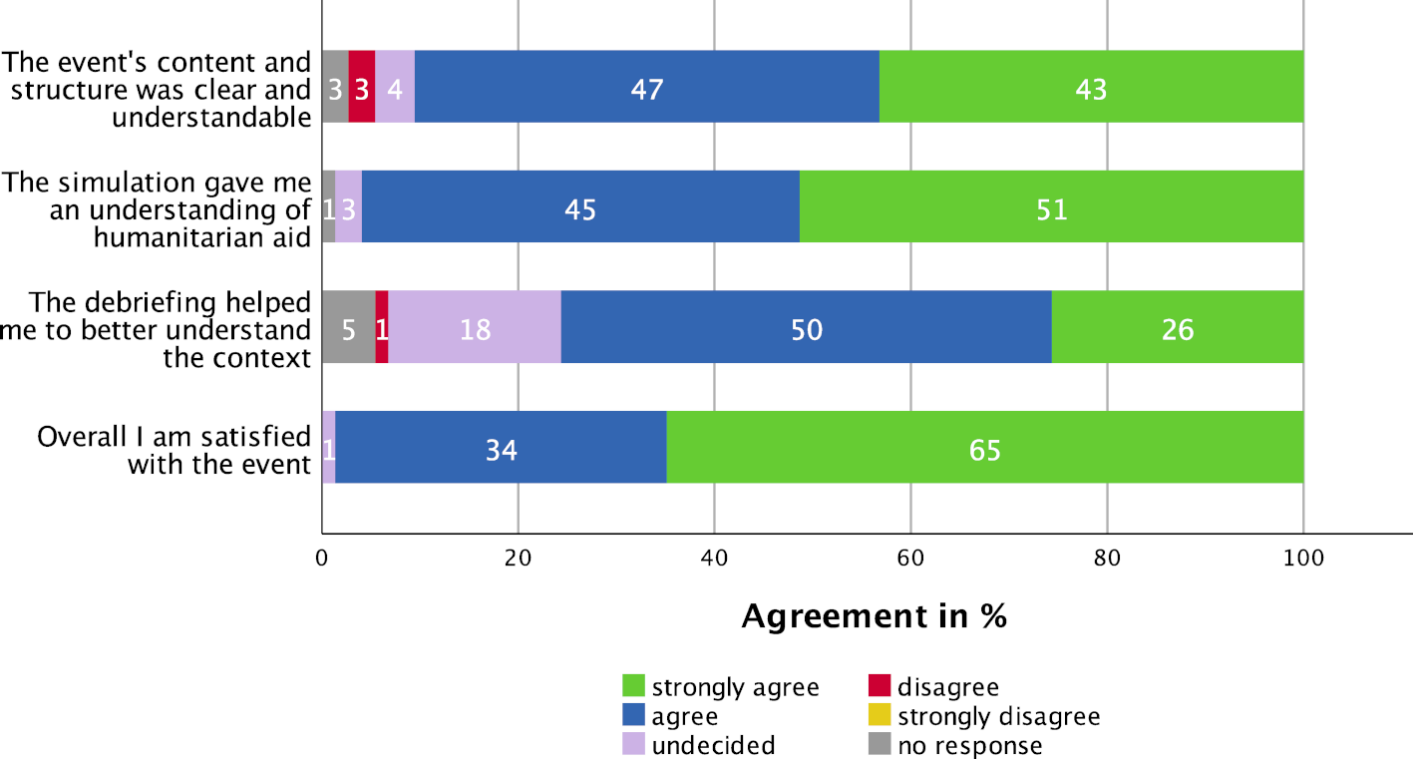
Agreement with four different statements regarding the workshop (n=74)

Agreement with various statements regarding the workshop structure, its components and overall participant satisfaction is shown in figure 1 (Fig. 1). The survey results indicated that participants found the game to involve an appropriate degree of difficulty and challenge. The level of difficulty of the simulation was rated as way too low by none of the participants, too low by 2.7%, appropriate by 90.5%, too high by 5.4% and way too high by 0%. 1.4% did not respond.

Free-text responses as well as oral feedback given by participants highlighted the need for small group sizes of 8-12 participants to ensure active participation and thus, satisfaction. Others suggested providing more theoretical background information on disaster medicine in the context of the simulation, for example about the structure and goals of different governmental and non-governmental actors. A small number of participants were overwhelmed by the complexity of the simulation and suggested a “test run” or distributing the rules beforehand.

## Discussion

The result of the workshops’ evaluation was very positive. A large majority of participants was overall satisfied with the event and all its components. Almost all participants found the level of difficulty to be appropriate. This is consistent with the findings of other AFTERSHOCK participant surveys, which we outlined in the project description [7], [8], [9], [10]. Although participants in these workshops came from very different contexts (WHO, military), they gave similarly positive ratings regarding their overall satisfaction, the level of complexity and the design of the game. The low-to-medium level of prior knowledge in our survey represents the sort of target audience for which AFTERSHOCK was designed. We saw very engaged participants during the workshops, with small group sizes and enough time for a proper introduction and debriefing being crucial to success. We disagree with the suggestion to distribute the rules beforehand or to perform a “test-run”. Experience in other settings mentioned above suggests that this is not necessarily very helpful: when players are provided the rules in advance they may feel a need to fully master them in advance. Introducing elements of the game as they become relevant during game play appears to work much better. Moreover, a limited degree of initial player confusion and uncertainty is also a valuable teaching tool: the immediate aftermath of a disaster, after all, is also characterized by uncertainty and limited information. Oral and written feedback also highlighted the importance of embedding the simulation within a more extensive course on disaster medicine to complement it with more theoretical background knowledge. Although we are confident that we achieved our main goal of providing our participants with a basic understanding of disaster medicine and humanitarian aid, especially regarding its complexities in practice, we agree with this assessment. It is also consistent with the scholarship on serious games, which stresses both the importance of integrating various course elements and value of debriefing sessions, which serve to highlight and contextualize games-based learning [11]. 

Such a course would have to be implemented at medical schools in Germany to comply with the concept for education on disaster medicine published in 2006 by the German “Schutzkommission”, a commission which advised the interior ministry on civil protection until 2015, the German Society for Disaster Medicine and the federal office for civil protection and disaster relief [12]. The concept includes 14 different topics and respective learning objectives, which, according to the commission, should be integrated into German medical education programs. The document is addressed to the German medical faculties. As previously mentioned, the German national competency-based catalogue of learning objectives for medicine (NKLM) currently includes only part of the proposed concept [http://www.nklm.de]. Both documents mainly focus on local and national settings and skills such as triage. In contrast, the simulation we used is set on both a much broader scale and the “meta level”, highlighting global challenges and the coordination between international organizations, governments and non-governmental actors. While the applicability of the specific scenario to the German context appears to be limited, these overarching aspects are certainly generalizable. This also applies to the soft skills which may be acquired during the simulation. Furthermore, we believe that medical students should be exposed to the challenges in other settings and environments.

Our project and its evaluation are limited by a selection bias: Participants chose to attend our workshops in their free time without any additional benefits such as credits being awarded. Participant’s ratings might be less positive if participation was mandatory. The validity of their responses regarding previous knowledge are limited by the self-assessment. Additionally, the evaluation form was only distributed at the end of the event. As mentioned before, the long-term learning effect of our workshops remains unclear as we did not conduct any follow-up evaluations. Furthermore, additional qualitative data (e.g. from interviews with participants) might have revealed benefits of the simulation in the realm of soft skills such as teamwork, management, effective communication etc., which contribute to the respective competencies outlined in the NKLM.

Another, more general risk with using simulations is the possibility of participants mainly focusing on utilizing game mechanics to win, thus neglecting the underlying principles [13]. This can negatively impact the learning effect and is especially important to consider when using digital games. Although these can be far more sophisticated than board games, they also bear the risk of obscuring underlying dynamics and key learning points through increased complexity and distracting visuals. Board games on the other hand use simplified, but clear game mechanics, thus facilitating discussion and encouraging critical insight. They are also easily modified and customized, even during a simulation. We thus consider AFTERSHOCK and similar simulations to be well-suited for use in medical education.

## Conclusion

Board games such as “AFTERSHOCK: A Humanitarian Crisis Game” are well-suited tools to simulate the complexity of humanitarian assistance. They provide opportunities to apply theoretical knowledge about disaster medicine in practice while experiencing the challenges of a dynamic environment. This and their high acceptance rate among students makes them suitable for use in medical education. To ensure long term learning, simulations should always be accompanied by theoretical coursework and effective debriefing.

## Acknowledgements

We would like to thank the German Medical Students’ Association for founding and funding the project.

## Note

Travel expenses that incurred in the context of the workshops conducted by SD and KG were reimbursed by the German Medical Students’ Association. Funds for acquiring the AFTERSHOCK board game were provided by the German Medical Students’ Association. RB designed the AFTERSHOCK board game, which is available for purchase. All profits from the sale of the game are donated to the World Food Programme and other UN humanitarian agencies. 

## Competing interests

The authors declare that they have no competing interests. 

## Supplementary Material

Evaluation form AFTERSHOCK-workshop
