# Supplementary material for: Crisis on the game board – a novel approach to teach medical students about disaster medicine
Source: GMS J Med Educ. 2018 Nov 15;35(4):Doc46. doi: 10.3205/zma001192 (PMC6278237; doi:10.3205/zma001192)
Supplement: Evaluation form AFTERSHOCK-workshop [file JME-35-46-s-001.pdf]

Evaluation form AFTERSHOCK-workshop

Semester	

I would describe my prior knowledge on humanitarian aid as…	very high	high	neither high nor low	low	very low

I have previously attended an event organized by the German Medical Students' Association	yes	no

The event's content structure was clear and understandable	strongly agree	agree	undecided	disagree	strongly disagree

The simulation gave me an understanding of humanitarian aid	strongly agree	agree	undecided	disagree	strongly disagree

The debriefing helped me to better understand the context	strongly agree	agree	undecided	disagree	strongly disagree

The level of difficulty of the event was...	way too low	too low	appropriate	too high	way too high

Overall I am satisfied with the event	strongly agree	agree	undecided	disagree	strongly disagree

Do you have other suggestions for improvement?	
